# Human Herpes Viruses Are Associated with Classic Fever of Unknown Origin (FUO) in Beijing Patients

**DOI:** 10.1371/journal.pone.0101619

**Published:** 2014-07-03

**Authors:** Weimin Zhou, Xinyi Tan, Yamin Li, Wenjie Tan

**Affiliations:** 1 Key Laboratory of Medical Virology, Ministry of Health; National Institute for Viral Disease Control and Prevention, China CDC, Beijing, China; 2 Beijing No. 2 High School, Xicheng, Beijing, China; Institute of Medicinal Biotechnology, Chinese Academy of Medical Sciences, China

## Abstract

**Background:**

Few reports have examined the viral aetiology of fever of unknown origin (FUO).

**Objective:**

This study determined the prevalence of human herpes virus (HHV) DNA in blood of Chinese patients with classic FUO using the polymerase chain reaction (PCR) and explored the possible role of HHV.

**Study design:**

Blood samples were collected from 186 patients (151 children, 35 adults) with classic FUO and 143 normal individuals in Beijing during the years 2009–2012. The HHV DNA, including Herpes simplex virus (HSV)-1/2, Varicella zoster virus (VZV), Cytomegalovirus (CMV), Epstein–Barr virus (EBV), and Human herpes virus (HHV)-6 and -7, was detected by multiplex PCR. The epidemiological and clinical features were also analysed.

**Results:**

HHV DNA was detected in 63 (33.9%) of the FUO patients, and the prevalence of EBV and HHV-6 was significantly higher than in the normal cohort. HHV co-infection was also frequent (10.2%) in the patients with FUO. The majority of patients with HHV infection present with a fever only. Our data also revealed that EBV infection was associated with hepatitis and abnormal blood indices, HHV-6 was associated with a cough, and HHV-7 was associated with hepatitis.

**Conclusions:**

HHVs are associated with Chinese patients (especially for children) with classic FUO. Our study adds perspective to the aetiological and clinical characteristics of classic FUO in beijing patients.

## Introduction

Fever is a common presenting complaint in children, accounting for nearly one-third of paediatric outpatient visits worldwide [1]. Fever of unknown origin (FUO) has occupied a special place within infectious diseases [2] since Petersdorf and Beeson first defined it in 1961 [3]. Although the original definition has been modified, the assessment of broad categories of illness (including infections, connective tissue disease, and malignancy) as causes of FUO remains useful [4]. The issue of FUO in paediatrics is hazy and represents a challenging diagnostic dilemma [5]. Presently, FUO cases are codified in four subclasses [6], [7]: classic, nosocomial, immune-deficient, and human immunodeficiency virus (HIV)-associated FUO. Infection is by far the most commonly identified aetiology of FUO in all paediatric studies [7], [8], including bacterial infections, brucellosis, tuberculosis, and typhoid fever. Viral infection, especially human herpes virus (HHV) infection, is also an important aetiological agent [9].

Studies of paediatric FUO to determine the relative incidence of different aetiologies suggest that aetiology varies geographically, economically, and according to the presence of vectors of infection and the availability of diagnostic tests [7]. Comparing developed and developing nations, infection is consistently the most common cause of FUO, but the types of infection vary [10]–[15]. Viral aetiologies for FUO are more common in developed countries [7], [13], particularly Epstein–Barr virus (EBV). It is disappointing that long-term outcome data are not available for the large number of children for whom no diagnosis for their FUO is established [7], [15].

### Objectives

Most of the available data are limited to large cohorts of paediatric patients, especially in China. This study investigated the prevalence of HHV DNA in blood using the polymerase chain reaction (PCR) in Chinese patients with classic FUO and explored its possible role.

## Study Design

### Ethical approval

All aspects of this study were performed in accordance with national ethics regulations and approved by the Institutional Review Board of the Centre for Disease Control and Prevention of China and the Ethics Committees of Beijing Hospital. The participants were informed regarding the study purpose and their right to confidentiality. Individual written informed consent was obtained from the parents or guardians of all participants.

### Patient enrolment and specimen collection

From January 2009 to December 2012, 329 blood specimens were collected from paediatric patients with classic FUO and normal children in the Beijing area. Classic FUO refers to the original classification by Petersdorf and Beeson. Our study applies the following definition for classic FUO: patients with a fever >101°F (38.3°C) of at least 8 days' duration, in whom no diagnosis is apparent after an initial outpatient or hospital evaluation that includes a careful history and physical examination and initial laboratory assessment. A comprehensive and meticulous history (*e*.*g*., illness of family members, recent visit to the tropics, and medication), repeated physical examination (*e*.*g*., skin rash, eschar, lymphadenopathy, or heart murmur), and myriad laboratory tests (serological, blood cultures, and immunological) were performed to diagnose all of the FUO patients. For example, blood and urine cultures and a chest radiograph were used to rule out typhoid fever, brucellosis or “septicaemia”, urinary tract infections, and pneumonia. A tuberculosis skin test was performed to rule out tuberculosis infections. All samples were negative for nuclear acid screening of HIV-1 and hepatitis B (HBV) and C (HCV) viruses. The serum alanine aminotransferase (ALT) and aspartate transaminase (AST) levels were used to evaluate liver function. No abscesses or solid tumours were detected using modern imaging techniques (*e*.*g*., ultrasonography, computed tomography (CT), and magnetic resonance imaging (MRI)) in any of the patients. Patients with lymphoma, drug fever, or necrotising lymphadenitis were excluded.

### Nucleic acid isolation and detection

Nucleic acid was extracted from a 200-µL blood sample using a QIAamp MinElute Virus Spin Kit (QIAGEN, Germany), according to the manufacturer's instructions. Multiplex PCR was performed as described [16], [17] for HHVs, including Herpes simplex virus (HSV)-1 and -2, Varicella zoster virus (VZV), Cytomegalovirus (CMV), EBV, and Human herpes virus (HHV)-6 and -7 (Table 1). A negative control (VTM only) in each set PCR assay was included to survey the possibility of laboratory contamination. All the methods were reported previously and validation in our lab [16]–[17].

**Table 1:** Primers(5′-3′) and Targets Used for the Detection of Human Herpes Viruses in the Study.

Viruses		Primer* and probe	Target and reaction
PCR			DNA POL
HSV1, 2	HSV-F	GCCAAGAAAAAGTACATCGGCGTCATC	1) 95°C for 10 min;
	HSV-R	TGAGGACAAAGTCCTGGATGTCCCTCT	2) Touchdown protocol
VZV	VZV-F	TCCGACATGCAGTCAATTTCAACGTC	10 cycles:
	VZV-R	GGTCGGGTAGACGCTACCACTCGTTT	95°C for 30 s
EBV	EBV-F	CTTAGAATGGTGGCCGGGCTGTAAAAT	70°C to 61°C
	EBV-R	ATCCAGTACGTCTTTGTGGAGCCCAAG	for 30 s with a 1°C decrease per cycle;
CMV	CMV-F	GCGCGTACCGTTGAAAGAAAAGCATAA	72°C for 1 min;
	CMV-R	TGGGCACTCGGGTCTTCATCTCTTYAC	3) 35 cycles:
HHV6	HHV6-F	ATGCGCCATCATAATGCTCGGATACA	95°C for 30 s; 60°C for 30 s; 72°C for 30 s
	HHV6-R	CCCTGCATTCTTACGGAAGCAAAACG	4) 72°C for 5 min
HHV7	HHV7-F	GCCCGTTTTCGGAAATATTGGAGAGAT	
	HHV7-R	ACGCACGAGACGCACTTTTCTTAAACA	

### Statistical analysis

The eligibility and classification of the clinical FUO syndromes were determined from the original record of each item in the medical history and an examination of the database. The impact of sexuality and age on the frequency distribution of HHVs between FUO and normal control children was analysed using the χ^2^ test and Fisher's exact test. All statistical analyses were performed with the Statistical Package for the Social Sciences (SPSS) ver. 17 (SPSS; Chicago, IL). Statistical significance was assessed using Tukey's test. *P*-values<0.05 were considered to be statistically significant.

## Results

### Study population

Blood samples were collected from 186 patients with classic FUO and 143 non-FUO normal children in the Beijing area between January 2009 and December 2011. The age and sex distributions are shown in Table 2. The mean age ± standard deviation of the 186 FUO patients was 9.16±13.59 years (range 43 days to 80 years). That of the 143 non-FUO children was 6.43±8.10 years (range 6 months to 32 years). Most of the FUO cases were younger than 15 years of age (151, 81.2%) and male (131, 70.4%).

**Table 2:** Demographic and aetiological data.

Parameter		Patients with FUO		Normal cohort without FUO		*P*-value
		No.	% (95% CI)	No.	% (95% CI)	
No. of cases		186		143		
Male/Female		131/55	70.4/29.6	77/66	53.8/46.2	
Mean age ±SD	(y) (range)	9.16±13.59 (43 days to 80 years)		6.43±8.10 (6 months to 32 years)		
Age	≤12 months	28	15.1	7	4.9	
	1–3 years	71	38.1	72	50.3	
	4–6 years	32	17.2	28	19.6	
	7–14 years	20	10.8	16	11.2	
	≥15 years	35	18.8	20	14.0	
HHV virus detected		63	33.9 (27.4–40.3)	32	22.4 (15.4–28.9)	0.023
HSV (1/2)		3	1.6 (0–3.8)	0	0	ND
VZV		5	2.7 (0.9–5.4)	0	0	ND
EBV		18	9.7 (6.5–15.1)	4	2.8 (0.7–5.9)	0.013
CMV		28	15.1 (10.2–19.9)	26	18.2 (12.3–25.2)	0.448
HHV-6		26	14.0 (10.2–20.1)	7	4.9 (1.4–8.7)	0.007
HHV-7		9	4.8 (2.7–8.1)	0	0	ND
Co-infection		19	10.2 (7.0–15.2)	5	3.5 (1.4–7.0)	0.020

### Frequency of HHV-infection in Chinese patients with classic FUO

**Figure 1:**
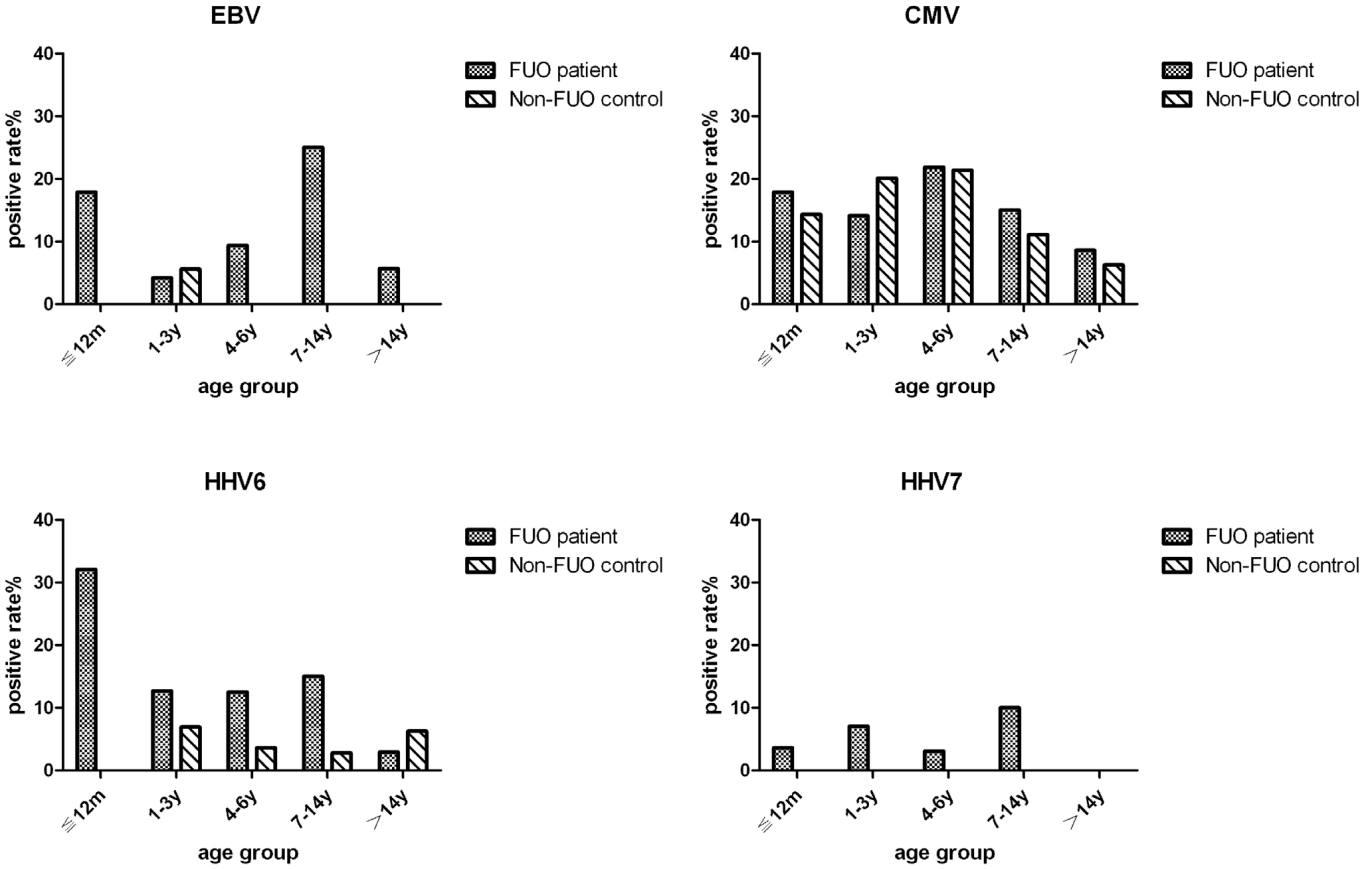
Distribution of HHVs among different age groups in this study.

From the blood samples, HHV DNA was identified in 63 (33.9%, 95% CI 27.4–40.3%) of the 186 FUO subjects and 32 (22.4%, 95% CI 15.4–28.9) of the 143 non-FUO controls (Table 2). In the 186 FUO blood samples tested, CMV (28, 15.1%), HHV-6 (26, 14%), and EBV (18, 9.7%) were the viruses detected most commonly, and the rates of infection were significantly higher than in the non-FUO group, except for CMV. In addition, HHV-7 (9, 4.8%), VZV (5, 2.7%), and HSV-1/2 (3, 1.6%) were detected in FUO cases, but not in non-FUO cases. The age distribution of the dominant HHVs (CMV, EBV, HHV-6, and HHV-7) among the FUO patients and normal controls is shown in Figure 1. Age had an impact on several HHVs (EBV, HHV-6, and HHV-7) in FUO. Both EBV and HHV-6 infection peaked in infants (younger than 12 months). HHV co-infection was also detected in 19 FUO cases (10.2%), which was significantly higher than in non-FUO cases (5, 3.5%) (Table 1).

### Clinical association with HHV-infection

The main clinical manifestation in the majority of FUO patients was fever only (n = 141, 75.81%). A few cases also showed signs of hepatitis (n = 16, 8.6%), abnormal blood indices (n = 16, 8.6%), and cough (n = 12, 6.45%), as shown in Table 3. Since we did not screen for all possible agents causing FUO, it was difficult to associate the clinical symptoms of the FUO patients with individual virus infections. However, some viral agents (EBV and HHV-7) were more strongly associated with hepatitis in FUO, as compared to HHV-6 infection (*p*<0.05). In addition, EBV infection was significantly more prevalent than other HHV infections among cases with an abnormal blood index (*p*<0.05). HHV-6 infection was more frequent among cases with a cough than other HHV infections. These data suggest that several HHV agents contribute to the occurrence of specific clinical symptoms among FUO cases.

**Table 3:** Association of clinical signs in the FUO patients (n = 184) with the viruses detected.

Aetiology detected	Fever only (n = 141)	+Hepatitis (n = 16)	+Abnormal blood indices (n = 16)	+Cough (n = 12)	*P*-value*
HHVs	46 (32.62%)	9 (56.25%)	3 (8.75%)	5 (41.67%)	0.139
EBV	11(7.8%)	4(25%)	2(12.5%)	1(8.3%)	**0.005**
CMV	20(14.1%)	5(31.25%)	0(0%)	1(8.3%)	0.07
HHV-6	22(15.6%)	0(0%)	1(6.25%)	3(25%)	**0.01**
HHV-7	4(2.8%)	3(18.75%)	1(6.25%)	1(8.3%)	**0.001**
Co-infection	12(8.51%)	4(25%)	1(6.25%)	2 (16.6%)	0.114

## Discussion

Despite a comprehensive examination and exhaustive workup in hospital, FUO remains a diagnostic and therapeutic challenge to expert physicians [2], [7], [8]. The three most common aetiological categories of FUO in children in order of frequency are infectious diseases, connective tissue diseases, and neoplasms [7]. Numerous studies have reported that a diagnosis is never established in 10∼26% of cases of FUO [2], [7], [15]. We applied the term FUO to patients with a fever >101°F (38.3°C) of at least 8-days' duration, in whom no diagnosis is apparent after an initial outpatient or hospital evaluation that includes a careful history and physical examination and initial laboratory assessment. To increase the knowledge of the aetiology of FUO, we screened blood samples from classic FUO cases in Beijing for HHV DNA. Our study first showed that HHVs were important aetiological agents among beijing patients (especially for children) with classic FUO.

Recently, Shi *et al*. analysed the clinical data for 997 adults with FUO admitted to Peking Union Medical College Hospital between January 2004 and October 2010 [15], and showed that infections were the most frequent cause of FUO and tuberculosis was the most important agent. However, no HHV DNA except CMV was screened and there were only 29 (3%) young patients (<20 yrs). Our study is the first report focusing on beijing paediatric patients with classic FUO that also screened for 7 HHV infections in blood samples from FUO patients and a normal cohort. CMV, HHV-6, and EBV were the viruses detected most commonly in FUO patients. The prevalence, co-infection, and clinical profiles in this study were consistent with similar studies of paediatric FUO patients [9], [13], [14]. Based on the data, our study clearly indicates an association between individual HHV infection and the clinical signs of FUO, which might be clinically important for diagnosis and treatment in the future.

The main limitation of our study is that it might be biased by the inclusion of a limited number of patients and exclusion criteria influenced by the sensitivity of diagnostic tests. Furthermore, we did not look specifically at non-classic FUO (nosocomial, human immunodeficiency virus related, or FUO in immunocompromised hosts). This study warrants a future non-anonymised investigation of the aetiology of FUO, to provide more comprehensive data on the course of infection and to establish the role of HHV in FUO, independently of other infectious agents, more clearly.
