# Correction: Human Herpes Viruses Are Associated with Classic Fever of Unknown Origin (FUO) in Beijing Patients

**DOI:** 10.1371/journal.pone.0110077

**Published:** 2014-09-29

**Authors:** The PLOS ONE Staff

There is an error in affiliation 2 for author Xinyi Tan. Affiliation 2 should be: Beijing No.2 High School, Dong Cheng District, Beijing, China.
